# 17β*H*-Periplogenin, a cardiac aglycone from the root bark of *Periploca sepium* Bunge

**DOI:** 10.1107/S1600536812018521

**Published:** 2012-05-02

**Authors:** Yu-Wei Zhang, Yong-Li Bao, Yin Wu, Chun-Lei Yu, Yu-Xin Li

**Affiliations:** a National Engineering Laboratory for Druggable Gene and Protein Screening, Northeast Normal University, ChangChun 130024, People’s Republic of China; b Institute of Genetics and Cytology, Northeast Normal University, ChangChun 130024, People’s Republic of China; c Research Center of Agriculture and Medicine Gene, Engineering of Ministry of Education, Northeast Normal University, ChangChun 130024, People’s Republic of China

## Abstract

The title compound {systematic name: 4-[(3*S*,5*S*,8*R*,9*S*,10*R*,13*R*,14*S*,17*S*)-3,5,14-trihy­droxy-10,13-dimethyl­hexa­deca­hydro-1*H*-cyclo­penta­[*a*]phenanthren-17-yl]furan-2(5*H*)-one}, C_23_H_34_O_5_, was isolated from the roots of *Periploca sepium* Bunge, a famous Chinese traditional herbal medicine. The three six-membered rings adopt chair conformations, the cyclo­pentane ring displays an approximate envelope conformation (with the C atom bearing the methyl substituent at the flap) and the five-membered lactone ring adopts an essentially planar [maximum deviation of 0.004 (8) Å] conformation. In the crystal, mol­ecules are linked into helical chains along [010] by O—H⋯O hydrogen bonds and weak C—H⋯O inter­actions. Two intra­molecular O—H⋯O hydrogen bonds are also present.

## Related literature

For the botanical and medicinal background to *Periploca sepium* Bunge, see: Li & Liu (2004 ▶); Yang *et al.* (2006 ▶). For the previous preparation and chemical structure determination of the title compound, see: Furuya *et al.* (1988 ▶); Kawaguchi *et al.* (1998 ▶).
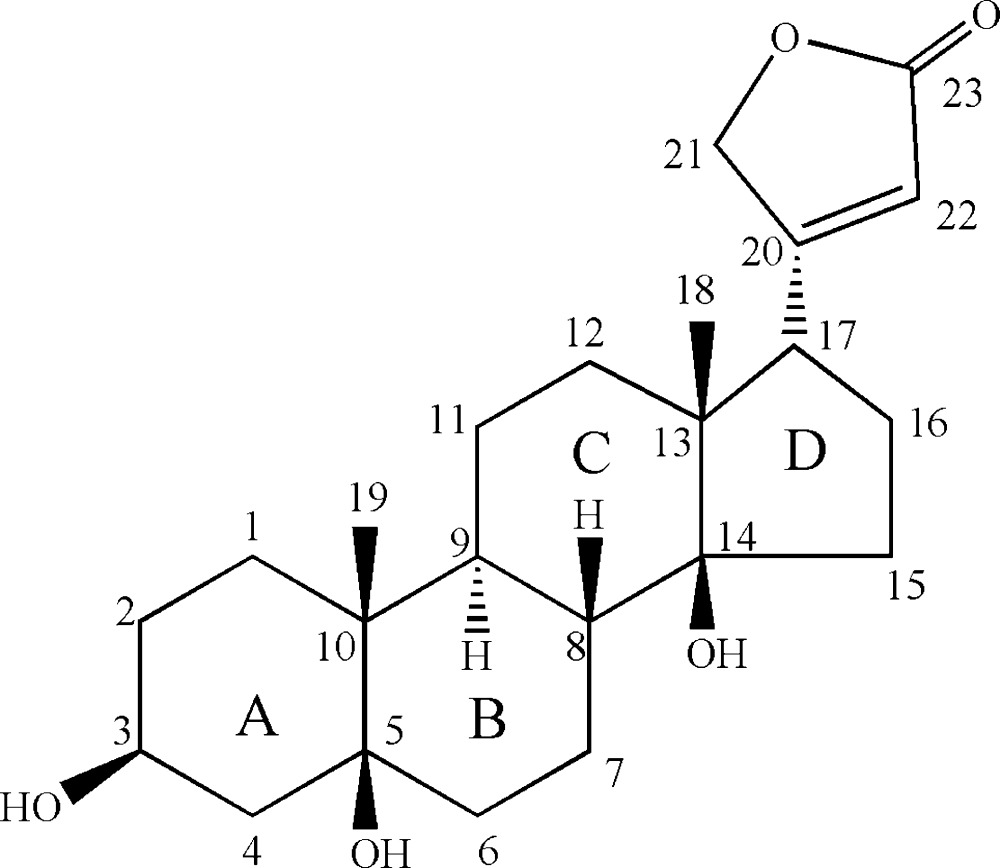


## Experimental

### 

#### Crystal data

C_23_H_34_O_5_
*M* *_r_* = 390.50
Monoclinic, 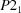

*a* = 7.434 (3) Å
*b* = 10.554 (4) Å
*c* = 13.537 (5) Å
β = 103.118 (5)°
*V* = 1034.4 (6) Å^3^
*Z* = 2
Mo *K*α radiation
μ = 0.09 mm^−1^
*T* = 296 K
0.26 × 0.24 × 0.02 mm

#### Data collection

Bruker APEX CCD area-detector diffractometer
Absorption correction: multi-scan (*SADABS*; Sheldrick, 1996 ▶) *T*_min_ = 0.978, *T*_max_ = 0.998
5262 measured reflections
1927 independent reflections
1097 reflections with *I* > 2σ(*I*)
*R*_int_ = 0.077

#### Refinement

*R*[*F* ^2^ > 2σ(*F* ^2^)] = 0.053
*wR*(*F* ^2^) = 0.126
*S* = 1.01
1927 reflections
254 parameters
1 restraint
H-atom parameters constrained
Δρ_max_ = 0.17 e Å^−3^
Δρ_min_ = −0.18 e Å^−3^

### 

Data collection: *SMART* (Bruker, 1997 ▶); cell refinement: *SAINT* (Bruker, 1999 ▶); data reduction: *SAINT*; program(s) used to solve structure: *SHELXS97* (Sheldrick, 2008 ▶); program(s) used to refine structure: *SHELXL97* (Sheldrick, 2008 ▶); molecular graphics: *SHELXTL* (Sheldrick, 2008 ▶); software used to prepare material for publication: *SHELXTL*.

## Supplementary Material

Crystal structure: contains datablock(s) I, global. DOI: 10.1107/S1600536812018521/wn2470sup1.cif

Structure factors: contains datablock(s) I. DOI: 10.1107/S1600536812018521/wn2470Isup2.hkl

Supplementary material file. DOI: 10.1107/S1600536812018521/wn2470Isup3.cml

Additional supplementary materials:  crystallographic information; 3D view; checkCIF report

## Figures and Tables

**Table 1:** Hydrogen-bond geometry (Å, °)

*D*—H⋯*A*	*D*—H	H⋯*A*	*D*⋯*A*	*D*—H⋯*A*
O1—H1*C*⋯O2	0.82	2.06	2.778 (5)	147
O2—H2*C*⋯O1	0.82	2.05	2.778 (5)	147
O3—H3*B*⋯O2^i^	0.82	2.16	2.977 (5)	175
C11—H11*A*⋯O5^ii^	0.97	2.57	3.393 (7)	143

Symmetry codes: (i) 
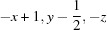
; (ii) 
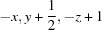
.

**Fig. 1.**
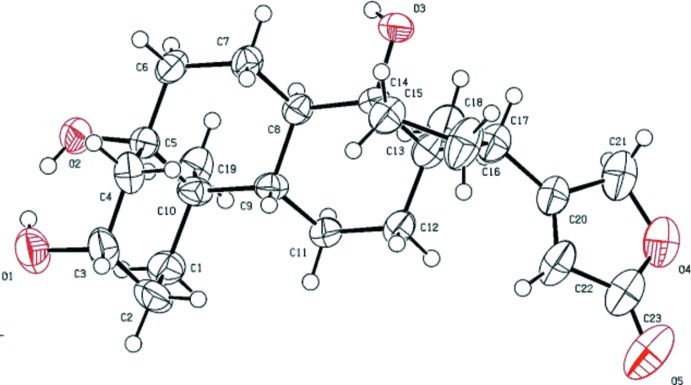
The molecular structure of the title compound, showing displacement ellipsoids at the 30% probability level.

**Fig. 2.**
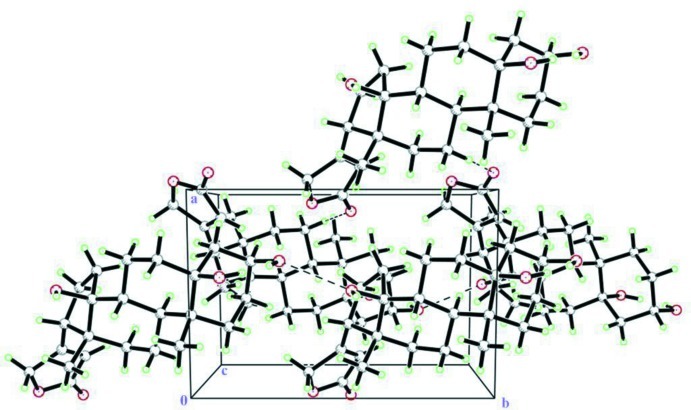
The crystal packing of the title compound, viewed along the c axis, showing [010] chains. Hydrogen bonds are shown as dashed lines.

## supplementary crystallographic information

### Comment

*Periploca sepium* Bunge (Asclepiadaceae) is native and widespread in the Loess hilly regions of northwest China (Li and Liu, 2004; Yang *et al.*, 2006). Its root bark, which is officially listed in the Chinese Pharmacopoeia under the name Cortex Periploca (Xiangjiapi in Chinese), has been frequently used to treat rheumatism and strengthen tendons and bones. The title compound, 17*β*H-periplogenin, which has been previously obtained as a biotransformation product of digitoxigenin (Furuya *et al.*, 1988; Kawaguchi *et al.*, 1998), was isolated from the root bark of *Periploca sepium* Bunge in our recent investigation. To the best of our knowledge, this is its first isolation from plant material. The isolated compound was identified by NMR spectra, which were compared with the previous report (Furuya *et al.*, 1988).

The crystal structure of 17*β*H-periplogenin has not yet been reported. In view of this, the crystal structure determination of the title compound was carried out and the results are presented here.

As shown in Fig. 1, the molecule consists of three six-membered rings (A, B and C), one cyclopentane ring (D) and one five-membered lactone ring. Rings A:B, B:C and C:D are *cis*-, *trans*- and *cis*- fused, respectively. The three six-membered rings adopt chair conformations, the cyclopentane ring displays an approximate envelope conformation with C13 as the flap atom, and the five-membered lactone ring adopts a planar conformation.

In the crystal structure, molecules are linked into helical chains along [010] through O3—H3B···O2 hydrogen bonds and C11—H11A···O5 weak interactions (Fig. 2 and Table 1). Two intramolecular O—H···O hydrogen bonds are also present (Table 1).

### Experimental

The air-dried and powdered roots of *Periploca sepium* Bunge (2.0 kg) were extracted with 70% EtOH (3×10 l, 3×2.0 h, 85 °C) under reflux conditions to give a crude extract, which was suspended in H_2_O and successively partitioned with CHCl_3_, EtOAc and *n*-butanol. A part of the CHCl_3_ fraction (50.0 g) was subjected to CC [SiO_2_, 200–300 mesh, CHCl_3_/MeOH (100:0, 95:5, 90:10, 80:20, 70:30, 60:40, 50:50 and 0:100(*v*/*v*)] to yield 8 fractions: Fr.1–8. Fr.3 was resubjected to CC (SiO_2_, 200–300 mesh, gradient of CHCl_3_/MeOH) and was further chromatographed on Sephadex LH-20 (CHCl_3_/MeOH, 1:1) to give 6 subfractions: SFr. 1–6. SFr. 4 (0.9 g) was then subjected to reverse phase preparative HPLC [Waters preparative HPLC system; XTERRA PREP MS C18 column, 5 µm, 19 mm × 150 mm; sample loading 30 - 60 mg/injection; the column was eluted with CH_3_OH/H_2_O system (52:48) at a flow rate of 16 ml/min] to provide the title compound (t_R_ = 4.81 min, 26 mg). ^1^H and ^13^C NMR spectroscopic data of this compound were recorded on a Bruker-AV-400 spectrometer, using CD_3_OD as solvent and Me_4_Si as internal standard. The stereochemistry was established by the X-ray diffraction experiment.

### Refinement

In the absence of significant anomalous scattering effects, Friedel pairs were merged. Initially all H-atoms were located in a difference Fourier map and at the last stage these H-atoms were geometrically treated.

The H-atoms were positioned geometrically (O—H = 0.82, C–H = 0.93–0.98 Å) and refined as riding with *U*iso(H) = *xU*eq(C, O), where *x* = 1.5 for methyl C and O and *x* = 1.2 for all other H-atoms.

### Crystal data

**Table:**

C_23_H_34_O_5_	*F*(000) = 424
*M**_r_* = 390.50	*D*_x_ = 1.254 Mg m^−^^3^
Monoclinic, *P*2_1_	Mo *K*α radiation, λ = 0.71073 Å
Hall symbol: P 2yb	Cell parameters from 5262 reflections
*a* = 7.434 (3) Å	θ = 1.5–25.0°
*b* = 10.554 (4) Å	µ = 0.09 mm^−^^1^
*c* = 13.537 (5) Å	*T* = 296 K
β = 103.118 (5)°	Block, colourless
*V* = 1034.4 (6) Å^3^	0.26 × 0.24 × 0.02 mm
*Z* = 2	

### Data collection

**Table:**

Bruker APEX CCD area-detector diffractometer	1927 independent reflections
Radiation source: fine-focus sealed tube	1097 reflections with *I* > 2σ(*I*)
Graphite monochromator	*R*_int_ = 0.077
ω scans	θ_max_ = 25.0°, θ_min_ = 1.5°
Absorption correction: multi-scan (*SADABS*; Sheldrick, 1996)	*h* = −8→8
*T*_min_ = 0.978, *T*_max_ = 0.998	*k* = −12→12
5262 measured reflections	*l* = −16→9

### Refinement

**Table:**

Refinement on *F*^2^	Secondary atom site location: difference Fourier map
Least-squares matrix: full	Hydrogen site location: inferred from neighbouring sites
*R*[*F*^2^ > 2σ(*F*^2^)] = 0.053	H-atom parameters constrained
*wR*(*F*^2^) = 0.126	*w* = 1/[σ^2^(*F*_o_^2^) + (0.0536*P*)^2^] where *P* = (*F*_o_^2^ + 2*F*_c_^2^)/3
*S* = 1.01	(Δ/σ)_max_ < 0.001
1927 reflections	Δρ_max_ = 0.17 e Å^−^^3^
254 parameters	Δρ_min_ = −0.18 e Å^−^^3^
1 restraint	Extinction correction: *SHELXL97* (Sheldrick, 2008), Fc^*^=kFc[1+0.001xFc^2^λ^3^/sin(2θ)]^-1/4^
Primary atom site location: structure-invariant direct methods	Extinction coefficient: 0.013 (3)

### Special details

**Table:**

Geometry. All e.s.d.'s (except the e.s.d. in the dihedral angle between two l.s. planes) are estimated using the full covariance matrix. The cell e.s.d.'s are taken into account individually in the estimation of e.s.d.'s in distances, angles and torsion angles; correlations between e.s.d.'s in cell parameters are only used when they are defined by crystal symmetry. An approximate (isotropic) treatment of cell e.s.d.'s is used for estimating e.s.d.'s involving l.s. planes.
Refinement. Refinement of *F*^2^ against ALL reflections. The weighted *R*-factor *wR* and goodness of fit *S* are based on *F*^2^, conventional *R*-factors *R* are based on *F*, with *F* set to zero for negative *F*^2^. The threshold expression of *F*^2^ > σ(*F*^2^) is used only for calculating *R*-factors(gt) *etc*. and is not relevant to the choice of reflections for refinement. *R*-factors based on *F*^2^ are statistically about twice as large as those based on *F*, and *R*- factors based on ALL data will be even larger.

### Fractional atomic coordinates and isotropic or equivalent isotropic displacement parameters (Å^2^)

**Table:**

	*x*	*y*	*z*	*U*_iso_*/*U*_eq_	
C1	0.3269 (8)	0.6136 (5)	0.1758 (4)	0.0561 (17)	
H1A	0.2076	0.5998	0.1916	0.067*	
H1B	0.3112	0.6777	0.1231	0.067*	
C2	0.4616 (9)	0.6639 (6)	0.2708 (4)	0.0639 (19)	
H2A	0.4156	0.7437	0.2908	0.077*	
H2B	0.4681	0.6042	0.3260	0.077*	
C3	0.6508 (9)	0.6834 (5)	0.2527 (4)	0.0555 (16)	
H3A	0.7350	0.7047	0.3174	0.067*	
C4	0.7198 (7)	0.5641 (5)	0.2117 (4)	0.0471 (15)	
H4A	0.7423	0.5004	0.2647	0.057*	
H4B	0.8368	0.5824	0.1944	0.057*	
C5	0.5873 (7)	0.5090 (5)	0.1180 (4)	0.0386 (13)	
C6	0.6653 (7)	0.3883 (5)	0.0839 (4)	0.0433 (14)	
H6A	0.7914	0.4036	0.0785	0.052*	
H6B	0.5937	0.3655	0.0170	0.052*	
C7	0.6629 (7)	0.2779 (5)	0.1564 (4)	0.0414 (14)	
H7A	0.7462	0.2959	0.2212	0.050*	
H7B	0.7058	0.2017	0.1290	0.050*	
C8	0.4684 (7)	0.2562 (5)	0.1723 (4)	0.0361 (13)	
H8A	0.3901	0.2376	0.1053	0.043*	
C9	0.3906 (7)	0.3778 (5)	0.2096 (4)	0.0373 (13)	
H9A	0.4724	0.4013	0.2745	0.045*	
C10	0.3898 (7)	0.4900 (5)	0.1345 (4)	0.0389 (13)	
C11	0.1987 (7)	0.3499 (5)	0.2297 (4)	0.0469 (15)	
H11A	0.1527	0.4252	0.2570	0.056*	
H11B	0.1136	0.3289	0.1663	0.056*	
C12	0.2059 (7)	0.2412 (5)	0.3039 (4)	0.0447 (15)	
H12A	0.0842	0.2290	0.3171	0.054*	
H12B	0.2899	0.2630	0.3675	0.054*	
C13	0.2699 (7)	0.1166 (5)	0.2639 (3)	0.0408 (14)	
C14	0.4603 (7)	0.1395 (5)	0.2376 (4)	0.0390 (13)	
C15	0.5971 (7)	0.1384 (5)	0.3422 (4)	0.0485 (15)	
H15A	0.7128	0.0996	0.3371	0.058*	
H15B	0.6219	0.2243	0.3673	0.058*	
C16	0.5062 (7)	0.0614 (7)	0.4146 (4)	0.069 (2)	
H16A	0.5827	−0.0105	0.4421	0.082*	
H16B	0.4876	0.1138	0.4703	0.082*	
C17	0.3218 (7)	0.0170 (6)	0.3500 (4)	0.0486 (15)	
H17A	0.3448	−0.0630	0.3182	0.058*	
C18	0.1250 (8)	0.0656 (6)	0.1759 (4)	0.0617 (18)	
H18A	0.0945	0.1293	0.1241	0.093*	
H18B	0.0163	0.0431	0.1990	0.093*	
H18C	0.1723	−0.0080	0.1487	0.093*	
C19	0.2521 (7)	0.4643 (6)	0.0324 (4)	0.0511 (16)	
H19A	0.2542	0.5339	−0.0129	0.077*	
H19B	0.1299	0.4551	0.0438	0.077*	
H19C	0.2866	0.3878	0.0030	0.077*	
C20	0.1787 (7)	−0.0093 (6)	0.4098 (4)	0.0473 (15)	
C22	0.1329 (8)	0.0576 (7)	0.4823 (4)	0.0612 (18)	
H22A	0.1868	0.1340	0.5074	0.073*	
C23	−0.0140 (9)	−0.0056 (7)	0.5170 (5)	0.0648 (19)	
C21	0.0617 (10)	−0.1253 (7)	0.3915 (6)	0.091 (2)	
H21A	−0.0109	−0.1279	0.3222	0.110*	
H21B	0.1366	−0.2014	0.4048	0.110*	
O5	−0.0980 (6)	0.0251 (6)	0.5800 (3)	0.0923 (17)	
O1	0.6546 (6)	0.7847 (4)	0.1820 (3)	0.0702 (13)	
H1C	0.6349	0.7560	0.1242	0.105*	
O2	0.5832 (5)	0.5977 (3)	0.0338 (2)	0.0470 (10)	
H2C	0.6038	0.6697	0.0563	0.071*	
O3	0.5070 (5)	0.0290 (3)	0.1852 (3)	0.0538 (11)	
H3B	0.4851	0.0431	0.1241	0.081*	
O4	−0.0578 (6)	−0.1125 (5)	0.4641 (4)	0.0856 (15)	

### Atomic displacement parameters (Å^2^)

**Table:**

	*U*^11^	*U*^22^	*U*^33^	*U*^12^	*U*^13^	*U*^23^
C1	0.057 (4)	0.052 (4)	0.067 (4)	0.022 (3)	0.028 (3)	0.015 (4)
C2	0.109 (6)	0.034 (3)	0.056 (4)	0.007 (3)	0.034 (4)	−0.007 (3)
C3	0.085 (5)	0.038 (3)	0.043 (4)	−0.013 (3)	0.013 (3)	0.004 (3)
C4	0.053 (4)	0.042 (4)	0.043 (3)	−0.011 (3)	0.005 (3)	0.006 (3)
C5	0.051 (3)	0.034 (3)	0.035 (3)	0.004 (3)	0.016 (3)	0.005 (3)
C6	0.041 (3)	0.051 (4)	0.042 (4)	0.002 (3)	0.019 (3)	0.006 (3)
C7	0.044 (3)	0.044 (3)	0.039 (3)	0.008 (3)	0.014 (3)	0.012 (3)
C8	0.042 (3)	0.039 (3)	0.029 (3)	0.001 (3)	0.011 (2)	0.002 (3)
C9	0.039 (3)	0.038 (3)	0.036 (3)	0.009 (3)	0.011 (2)	−0.001 (3)
C10	0.045 (3)	0.038 (3)	0.033 (3)	0.013 (3)	0.008 (2)	0.002 (3)
C11	0.052 (4)	0.039 (3)	0.058 (4)	0.008 (3)	0.030 (3)	0.005 (3)
C12	0.047 (4)	0.048 (4)	0.043 (3)	0.000 (3)	0.020 (3)	0.005 (3)
C13	0.045 (3)	0.049 (4)	0.028 (3)	0.000 (3)	0.006 (3)	0.003 (3)
C14	0.048 (3)	0.037 (3)	0.038 (3)	0.005 (3)	0.022 (3)	−0.001 (3)
C15	0.042 (3)	0.058 (4)	0.045 (3)	0.005 (3)	0.011 (3)	0.008 (3)
C16	0.043 (4)	0.099 (5)	0.062 (4)	−0.004 (4)	0.008 (3)	0.031 (4)
C17	0.046 (3)	0.056 (4)	0.046 (3)	0.000 (3)	0.014 (3)	0.010 (3)
C18	0.066 (4)	0.070 (4)	0.046 (3)	−0.020 (4)	0.007 (3)	−0.004 (3)
C19	0.043 (3)	0.067 (4)	0.040 (3)	0.001 (3)	0.003 (3)	0.017 (3)
C20	0.041 (3)	0.053 (4)	0.047 (4)	−0.001 (3)	0.011 (3)	0.012 (3)
C22	0.059 (4)	0.084 (5)	0.045 (3)	−0.020 (4)	0.021 (3)	0.004 (4)
C23	0.053 (4)	0.089 (6)	0.054 (4)	−0.007 (4)	0.017 (3)	0.013 (5)
C21	0.106 (6)	0.074 (5)	0.111 (6)	−0.025 (5)	0.060 (5)	0.006 (5)
O5	0.071 (3)	0.150 (5)	0.060 (3)	−0.008 (3)	0.025 (2)	0.018 (4)
O1	0.116 (4)	0.046 (2)	0.052 (3)	−0.013 (2)	0.025 (3)	0.002 (2)
O2	0.063 (2)	0.043 (2)	0.037 (2)	−0.0012 (19)	0.0163 (18)	0.0047 (19)
O3	0.083 (3)	0.038 (2)	0.051 (2)	0.014 (2)	0.036 (2)	0.003 (2)
O4	0.088 (4)	0.072 (3)	0.107 (4)	−0.024 (3)	0.044 (3)	0.006 (3)

### Geometric parameters (Å, º)

**Table:**

C1—C10	1.533 (7)	C12—H12A	0.9700
C1—C2	1.534 (8)	C12—H12B	0.9700
C1—H1A	0.9700	C13—C18	1.512 (7)
C1—H1B	0.9700	C13—C17	1.552 (7)
C2—C3	1.496 (8)	C13—C14	1.555 (6)
C2—H2A	0.9700	C14—O3	1.448 (6)
C2—H2B	0.9700	C14—C15	1.545 (7)
C3—O1	1.440 (6)	C15—C16	1.542 (7)
C3—C4	1.512 (8)	C15—H15A	0.9700
C3—H3A	0.9800	C15—H15B	0.9700
C4—C5	1.532 (7)	C16—C17	1.523 (7)
C4—H4A	0.9700	C16—H16A	0.9700
C4—H4B	0.9700	C16—H16B	0.9700
C5—O2	1.469 (6)	C17—C20	1.502 (7)
C5—C6	1.515 (7)	C17—H17A	0.9800
C5—C10	1.548 (7)	C18—H18A	0.9600
C6—C7	1.527 (7)	C18—H18B	0.9600
C6—H6A	0.9700	C18—H18C	0.9600
C6—H6B	0.9700	C19—H19A	0.9600
C7—C8	1.527 (7)	C19—H19B	0.9600
C7—H7A	0.9700	C19—H19C	0.9600
C7—H7B	0.9700	C20—C22	1.315 (7)
C8—C14	1.525 (7)	C20—C21	1.490 (8)
C8—C9	1.539 (6)	C22—C23	1.447 (8)
C8—H8A	0.9800	C22—H22A	0.9300
C9—C11	1.540 (6)	C23—O5	1.210 (7)
C9—C10	1.560 (6)	C23—O4	1.335 (8)
C9—H9A	0.9800	C21—O4	1.474 (8)
C10—C19	1.546 (7)	C21—H21A	0.9700
C11—C12	1.517 (7)	C21—H21B	0.9700
C11—H11A	0.9700	O1—H1C	0.8200
C11—H11B	0.9700	O2—H2C	0.8200
C12—C13	1.538 (7)	O3—H3B	0.8200
			
C10—C1—C2	113.9 (4)	C11—C12—H12A	109.1
C10—C1—H1A	108.8	C13—C12—H12A	109.1
C2—C1—H1A	108.8	C11—C12—H12B	109.1
C10—C1—H1B	108.8	C13—C12—H12B	109.1
C2—C1—H1B	108.8	H12A—C12—H12B	107.9
H1A—C1—H1B	107.7	C18—C13—C12	111.1 (5)
C3—C2—C1	111.7 (5)	C18—C13—C17	111.2 (4)
C3—C2—H2A	109.3	C12—C13—C17	111.1 (4)
C1—C2—H2A	109.3	C18—C13—C14	113.4 (4)
C3—C2—H2B	109.3	C12—C13—C14	108.6 (4)
C1—C2—H2B	109.3	C17—C13—C14	101.1 (4)
H2A—C2—H2B	107.9	O3—C14—C8	108.7 (3)
O1—C3—C2	111.8 (5)	O3—C14—C15	105.2 (4)
O1—C3—C4	108.3 (4)	C8—C14—C15	115.7 (4)
C2—C3—C4	110.8 (5)	O3—C14—C13	108.6 (4)
O1—C3—H3A	108.6	C8—C14—C13	114.5 (4)
C2—C3—H3A	108.6	C15—C14—C13	103.5 (4)
C4—C3—H3A	108.6	C16—C15—C14	107.1 (4)
C3—C4—C5	114.3 (5)	C16—C15—H15A	110.3
C3—C4—H4A	108.7	C14—C15—H15A	110.3
C5—C4—H4A	108.7	C16—C15—H15B	110.3
C3—C4—H4B	108.7	C14—C15—H15B	110.3
C5—C4—H4B	108.7	H15A—C15—H15B	108.5
H4A—C4—H4B	107.6	C17—C16—C15	105.3 (4)
O2—C5—C6	103.9 (4)	C17—C16—H16A	110.7
O2—C5—C4	107.2 (4)	C15—C16—H16A	110.7
C6—C5—C4	110.6 (4)	C17—C16—H16B	110.7
O2—C5—C10	110.0 (4)	C15—C16—H16B	110.7
C6—C5—C10	112.1 (4)	H16A—C16—H16B	108.8
C4—C5—C10	112.5 (4)	C20—C17—C16	113.7 (4)
C5—C6—C7	112.6 (4)	C20—C17—C13	116.4 (4)
C5—C6—H6A	109.1	C16—C17—C13	105.1 (4)
C7—C6—H6A	109.1	C20—C17—H17A	107.0
C5—C6—H6B	109.1	C16—C17—H17A	107.0
C7—C6—H6B	109.1	C13—C17—H17A	107.0
H6A—C6—H6B	107.8	C13—C18—H18A	109.5
C6—C7—C8	110.8 (4)	C13—C18—H18B	109.5
C6—C7—H7A	109.5	H18A—C18—H18B	109.5
C8—C7—H7A	109.5	C13—C18—H18C	109.5
C6—C7—H7B	109.5	H18A—C18—H18C	109.5
C8—C7—H7B	109.5	H18B—C18—H18C	109.5
H7A—C7—H7B	108.1	C10—C19—H19A	109.5
C14—C8—C7	111.5 (4)	C10—C19—H19B	109.5
C14—C8—C9	114.6 (4)	H19A—C19—H19B	109.5
C7—C8—C9	111.1 (4)	C10—C19—H19C	109.5
C14—C8—H8A	106.4	H19A—C19—H19C	109.5
C7—C8—H8A	106.4	H19B—C19—H19C	109.5
C9—C8—H8A	106.4	C22—C20—C21	109.0 (5)
C8—C9—C11	109.3 (4)	C22—C20—C17	129.6 (6)
C8—C9—C10	111.4 (4)	C21—C20—C17	121.3 (6)
C11—C9—C10	113.2 (4)	C20—C22—C23	109.7 (6)
C8—C9—H9A	107.5	C20—C22—H22A	125.2
C11—C9—H9A	107.5	C23—C22—H22A	125.2
C10—C9—H9A	107.5	O5—C23—O4	120.1 (7)
C1—C10—C19	106.3 (4)	O5—C23—C22	130.8 (8)
C1—C10—C5	108.6 (4)	O4—C23—C22	109.0 (6)
C19—C10—C5	110.6 (4)	O4—C21—C20	103.3 (6)
C1—C10—C9	111.4 (4)	O4—C21—H21A	111.1
C19—C10—C9	110.7 (4)	C20—C21—H21A	111.1
C5—C10—C9	109.2 (4)	O4—C21—H21B	111.1
C12—C11—C9	111.5 (4)	C20—C21—H21B	111.1
C12—C11—H11A	109.3	H21A—C21—H21B	109.1
C9—C11—H11A	109.3	C3—O1—H1C	109.5
C12—C11—H11B	109.3	C5—O2—H2C	109.5
C9—C11—H11B	109.3	C14—O3—H3B	109.5
H11A—C11—H11B	108.0	C23—O4—C21	108.9 (5)
C11—C12—C13	112.4 (4)		

### Hydrogen-bond geometry (Å, º)

**Table:**

*D*—H···*A*	*D*—H	H···*A*	*D*···*A*	*D*—H···*A*
O1—H1*C*···O2	0.82	2.06	2.778 (5)	147
O2—H2*C*···O1	0.82	2.05	2.778 (5)	147
O3—H3*B*···O2^i^	0.82	2.16	2.977 (5)	175
C11—H11*A*···O5^ii^	0.97	2.57	3.393 (7)	143

Symmetry codes: (i) −*x*+1, *y*−1/2, −*z*; (ii) −*x*, *y*+1/2, −*z*+1.

## References

[bb1] Bruker (1997). *SMART* Bruker AXS Inc., Madison, Wisconsin, USA.

[bb2] Bruker (1999). *SAINT* Bruker AXS Inc., Madison, Wisconsin, USA.

[bb3] Furuya, T., Kawaguchi, K. & Hirotani, M. (1988). *Phytochemistry*, **27**, 2129–2133.

[bb4] Kawaguchi, K., Koike, S., Hirotani, M., Fujihara, M., Furuya, T., Iwata, R. & Morimoto, K. (1998). *Phytochemistry*, **47**, 1261–1265. 10.1016/s0031-9422(97)00748-6 9611827

[bb5] Li, S. W. & Liu, L. P. (2004). *Acta Bot. Boreali-Occidentalia Sin.* **24**, 275–280.

[bb6] Sheldrick, G. M. (1996). *SADABS* University of Göttingen, Germany.

[bb7] Sheldrick, G. M. (2008). *Acta Cryst.* A**64**, 112–122. 10.1107/S0108767307043930 18156677

[bb8] Yang, C. H., Wang, Y. Y., Zhou, Z. F. & Zhang, G. C. (2006). *For. Res.* **19**, 231–234.

